# Upregulation of Circular RNA CircNFIB Attenuates Cardiac Fibrosis by Sponging miR-433

**DOI:** 10.3389/fgene.2019.00564

**Published:** 2019-06-20

**Authors:** Yujiao Zhu, Wen Pan, Tingting Yang, Xiangmin Meng, Zheyi Jiang, Lichan Tao, Lijun Wang

**Affiliations:** ^1^Cardiac Regeneration and Ageing Lab, Institute of Cardiovascular Sciences, School of Life Sciences, Shanghai University, Shanghai, China; ^2^Department of Cardiology, The Third Affiliated Hospital of Soochow University, Changzhou, China

**Keywords:** cardiac fibrosis, circNFIB, miR-433, primary adult cardiac fibroblasts, transforming growth factor-β

## Abstract

Cardiac fibrosis is the pathological consequence of fibroblast proliferation and fibroblast-to-myofibroblast transition. As a new class of endogenous non-coding RNAs, circular RNAs (circRNAs) have been identified in many cardiovascular diseases including fibrosis, generally acting as microRNA (miRNA) sponges. Here, we report that the expression of circRNA–circNFIB was decreased in mice post-myocardial infarction heart samples, as well as in primary adult cardiac fibroblasts treated with TGF-β. Forced expression of circNFIB decreased cell proliferation in both NIH/3T3 cells and primary adult fibroblasts as evidenced by EdU incorporation. Conversely, inhibition of circNFIB promoted adult fibroblast proliferation. Furthermore, circNFIB was identified as a miR-433 endogenous sponge. Overexpression of circNFIB could attenuate pro-proliferative effects induced by the miR-433 mimic while inhibition of circNFIB exhibited opposite results. Finally, upregulation of circNFIB also reversed the expression levels of target genes and downstream signaling pathways of miR-433. In conclusion, circNFIB is critical for protection against cardiac fibrosis. The circNFIB–miR-433 axis may represent a novel therapeutic approach for treatment of fibrotic diseases.

## Introduction

Cardiac fibrosis, defined as the imbalance of extracellular matrix (ECM) production and degradation, which contributes to accumulation of connective tissue proteins in the interstitial and perivascular tissues, plays a crucial role in the development and evolution of heart failure (Weber et al., 2013). Fibrosis is a common pathological feature of most adverse ventricular remodeling, such as myocardial infarction (MI), diabetic cardiomyopathy, hypertrophic, and dilated cardiomyopathy (DCM) (Gyongyosi et al., 2017). It normally includes three overlapping phases: proliferation, granulation, and maturation. Cardiac fibroblasts are the predominant cells involved in each of these phases (Lajiness and Conway, 2014). Under pathological conditions, increased mechanical stress and release of cytokines and growth factors dynamically modulate fibroblast proliferation and *trans*-differentiation into highly proliferative transformed fibroblasts, termed myofibroblasts. The transformation involves subcellular changes such as increased expression of α-smooth muscle actin (α-SMA) and secretion of extracellular procollagen chains into collagen type I and type III fibrils (Li et al., 2014; Watson et al., 2014; Gyongyosi et al., 2017). The accumulation of excessive diffused collagen results in disruption of myocardial architecture, myofibrils disarray, and mechanical, electrical, and vasomotor dysfunction (Segura et al., 2014).

Clinical studies have shown that cardiac fibrosis is closely related with higher long-term mortality in patients with cardiac diseases, particularly for those with heart failure (Besler et al., 2017; Bacmeister et al., 2019). Thus, detecting, preventing, and treating myocardial fibrosis have critical significance in the strategies of heart failure therapy. Based on microRNA (miRNA) array, we have previously reported different expression of miRNAs during MI remodeling. In particular, miR-433 was highly conserved and was not only significantly increased in MI remodeling samples but also upregulated in a rodent model of doxorubicin-induced cardiomyopathy and human DCM. Furthermore, upregulation of miR-433 increased proliferation and *trans*-differentiation of cardiac fibroblasts into myofibroblasts, whereas knockdown of miR-433 suppressed these responses depending on the stimulation of transforming growth factor-β (TGF-β). More importantly, treatment with miR-433 antagomir or adeno-associated virus 9 (AAV9)-mediated miR-433 sponge reversed cardiac dysfunction and fibrosis induced by MI remodeling (Tao et al., 2016). Thus, inhibition of miR-433 is regarded as a novel strategy for the treatment of cardiac fibrosis.

Circular RNAs (circRNAs) are a new class of endogenous non-coding RNAs with single-stranded, covalently closed structures that are produced by back splicing (Hentze and Preiss, 2013; Chen and Yang, 2015). Lacking 5′- to 3′- polarity and polyadenylated tails, circRNAs are conserved and stable, and numerous circRNAs seem to be specifically expressed in a given cell type or developmental phase (Jeck et al., 2013; Memczak et al., 2013). Natural endogenous circRNAs can contain conserved miRNA target sites, thus functioning as efficient miRNA sponges to play important roles in many physiological and pathological processes (Wang et al., 2016; Hall et al., 2018). Therefore, studies on circRNAs have provided promising new insights into miRNA regulation.

Our previous study showed that miR-433 inhibition could improve post-MI cardiac function and fibrosis. In the current study, we report that circRNA circNFIB (circBase: mmu_circ_0011794) could function as a miR-433 endogenous sponge to regulate cardiac fibrosis. We firstly generated circNFIB overexpression construct and designed siRNA of circNFIB, identifying that knockdown of circNFIB increased proliferation of primary adult cardiac fibroblasts, whereas overexpression of circNFIB reversed cell proliferation in both NIH/3T3 cell lines and adult cardiac fibroblasts upon TGF-β stimulation. Luciferase assay and functional rescue experiments further confirmed that circNFIB regulated cardiac fibrosis by directly targeting miR-433. Collectively, our findings indicate that circNFIB could act as an endogenous miR-433 sponge to inhibit fibroblast proliferation and therefore might be regarded as a potential therapy for the treatment of cardiac fibrosis.

## Materials and Methods

### Construction of circRNA

A double-stranded DNA PCR product was amplified and then cloned into a commercially available circRNA expression vector pLO-ciR vector (Guangzhou Geneseed Biotech Co., China). Plasmid DNA was extracted and subsequently sequenced. To verify the circNFIB overexpression vector (circNFIB OE), we next designed a divergent primer that specifically amplified the back-spliced forms of circNFIB, and the cross-junction fragments were verified by Sanger sequence. After confirming the results of sequence, the circNFIB OE was successfully generated. The empty vector Plo-ciR was used as negative control (NC circNFIB).

### Quantitative Real-Time Polymerase Chain Reactions

Total RNAs were extracted from heart samples and cardiac fibroblasts by using RNAiso Plus (Takara) according to the manuscript’s instructions. Total RNAs (800 ng, 20 μl system) were reverse transcribed using a cDNA synthesis kit (Bio-Rad, Hercules, CA, United States). Then, quantification of circRNA and mRNA was performed using SYBR Premix Ex TaqTM (Takara) and the Roche Real-Time PCR Detection System (Roche). 18S RNA was used as an internal control for gene expressions. For quantitative miRNA analysis, the Bulge-Loop miRNA qPCR primer kit (RiboBio, Guangzhou, China) was used to determine the expression levels of miRNA, and the levels of small nuclear U6 were used to normalize the miRNA expression level. The primer sequences of circNFIB were as follows—forward: TGAACGAGATCAAGCACCCAT; reverse: CTGCTCGGTGGAGAAGACAG. For 18S: forward: TCAAGAACGAAAGTCGGAGG; reverse: GACATCTAAGGGCATCAC.

### Isolation of Mice Cardiac Fibroblasts

Cardiac fibroblasts were isolated for each round of experiments from 8- to 10-week male adult C57BL/6N mice by trypsin-collagenase-based digestion and pooled before pre-plating and culturing. Briefly, heart samples were explanted and washed in ice-cold Hanks medium. After dicing into small pieces and further washing in ice-cold Hanks medium for removal of plasma contaminants, the cardiac pieces were pre-digested for 10 min at 37°C in an enzyme solution (60% trypsin and 40% collagenase dissolved in Hanks medium). After replacement of the enzyme solution, the cardiac pieces were incubated at 37°C for 7 min for 6–7 cycles. Fibroblast-containing supernatants were diluted with HS, centrifuged at 1,000 rpm for 5 min, and pooled together in culture medium [DMEM 4.5 g/l glucose, 10% fetal bovine serum (FBS), 100 U/ml penicillin, and 100 μg/ml streptomycin] in a 5% CO_2_ incubator. All the pooled supernatants were filtered through a 100-μm cell strainer and pre-plated at 37°C in a humidified atmosphere of 5% CO_2_ for 48 h. Cells at passage 3 were used in all experiments shown.

### Transfection of Synthetic circRNA and Small Interfering RNA

All transfections and assays were performed on cardiac fibroblasts at passage 3 or NIH/3T3 cell lines cultured in low serum medium (1% FBS). CircNFIB is knocked down using custom-designed small interfering RNA (siRNA) oligonucleotides and, additionally, a targeting site across the head-to-tail junction. CircNFIB siRNA target sequence is 5′-GAGATCAAGCACCCATAAC-3′ (purchased from Biotend, Shanghai, China). A non-related, scrambled siRNA sequence is used as a control (5′-TTCTCCGAACGTGTCACGT-3′). Cardiac fibroblasts or NIH/3T3 cell lines were exposed to either plasmid overexpressing circNFIB versus empty vector (50 nM) or siRNA for circNFIB versus negative control (NC, 50 nM) for 48 h, and treated with recombinant human TGF-β1 (10 ng/ml, Sino Biological, Beijing, China) for 24 h. Notably, considering the low expression abundance of circNFIB in adult cardiac fibroblast, plasmid overexpressing circNFIB was first transfected and then cells were exposed to circNFIB siRNA versus NC. miR-433 mimic and NC (50 nM) were purchased from RiboBio. All the transfections were carried out by using Lipofectamine RNAiMAX Transfection Reagent (Invitrogen).

### Establishment of MI Remodeling Model

Animal procedures were performed in C57BL/6N male mice (8–10 weeks old). Following full anesthesia through the intraperitoneal injection of 3% chloral hydrate, the mice were intubated and placed on a standard rodent ventilator. An incision was made between the third and fourth intercostal space, and MI mode was generated by ligating the left anterior descending coronary artery (LAD) using 7-0 suture. The sham group was created by the same process but without LAD ligation.

### Immunofluorescence and EdU Staining

Forty-eight hours after transfection, cardiac fibroblasts or NIH/3T3 cells were fixed with 4% paraformaldehyde (PFA) for 20 min at room temperature. After washing three times with phosphate-buffered saline (PBS), cells were permeabilized with 0.2% Triton X-100 (PBST) for 20 min at room temperature. Then, cardiac fibroblasts were blocked with 10% goat serum in PBST for 1 h at room temperature. Primary antibodies with α-SMA-Cy3 (1:100, Sigma, St. Louis, MO, United States) diluted in 10% goat serum were incubated at 4°C overnight. Click-iT Plus EdU Alexa Fluor 488 Imaging Kit (KeyGEN BioTECH, Nanjing, China) was used to detect proliferation according to the manufacturer’s instructions. Nuclei were counterstained with DAPI. The proliferative rate was calculated by the number of EdU-positive cells/DAPI.

### Luciferase Reporter Assay

A luciferase reporter vector containing miR-433 binding site was constructed by inserting annealed miR-433 binding site into the pMIRGLO vector (Promega, Madison, WI, United States) using PmeI and XbaI restriction enzymes. When the cell confluence reached about 80%, the miR-433 mimic (RiboBio) and pMIR-circNFIB-wide type or pMIR-circNFIB-mutant were co-transfected into HEK293T cells using Lipofectamine 2000 (Investigen). After 48 h of co-transfection, the luciferase activity was measured with a dual luciferase reporter assay system (Promega). The relative fold change of luciferase activity normalized with NC.

### Western Blot Analysis

The total proteins were extracted from cardiac fibroblasts using protein lysis buffer (RIPA) containing 1 mM PMSF (KeyGEN BioTECH, Nanjing, China). Then, 30 μg of total proteins was loaded and separated on 10% sodium dodecyl sulfate-polyacrylamide gel electrophoresis (SDS-PAGE) gels. After electrophoresis, the proteins were transferred to polyvinylidene fluoride (PVDF) membrane and then blocked with 5% skim milk in tris saline with tween 20 (TBST) for 1 h at room temperature. The PVDF membrane was then incubated with primary antibodies at 4°C overnight. The primary antibodies used were from the following sources: AZIN1 (1:500, cat. NO. 11548, Proteintech), Jnk1 (1:1,000, cat. NO. 3708, Cell Signaling Technology, Boston, MA, United States), p38 (1:1,000, cat. NO. 8690, CST), p-p38 (1:1,000, cat. NO. 4511, CST), ERK (1:1,000, cat. NO. 4695, CST), p-ERK (1:1,000, cat. NO. 4370, CST), Smad3 (1:1,000, cat. NO. 9523, CST), p-Smad3 (1:1,000, cat. NO. 9520, CST), and GAPDH (1:1,000, cat. NO. AP0063, Bioworld). The PVDF membrane was washed three times with TBST and then incubated with a second antibody, which was horseradish peroxidase (HRP)-labeled anti-mouse or anti-rabbit immunoglobulin G (IgG; 1:1,000, CST). All the proteins were visualized by ECL chemiluminescence kit (Thermo Fisher, Waltham, MA, United States), and the quantification of each band was performed using Imagelab Software (Bio-Rad) as compared to GAPDH. Uncropped images of western blots are available in Supplementary Figure S1.

### Statistical Analysis

Data were presented at mean of independent experiments/independent samples ± SE. Statistical analysis was performed using Prism Software (GraphPad Prism 5). For analysis of two groups, Student’s *t*-test was used; for comparison of three or more groups, one-way ANOVA followed by Bonferroni’s post test was applied. A *p-*value less than 0.05 was considered to be statistically different.

## Results

### CircNFIB Is Downregulated in Cardiac Fibrosis *in vivo* and *in vitr*o

**FIGURE 1:**
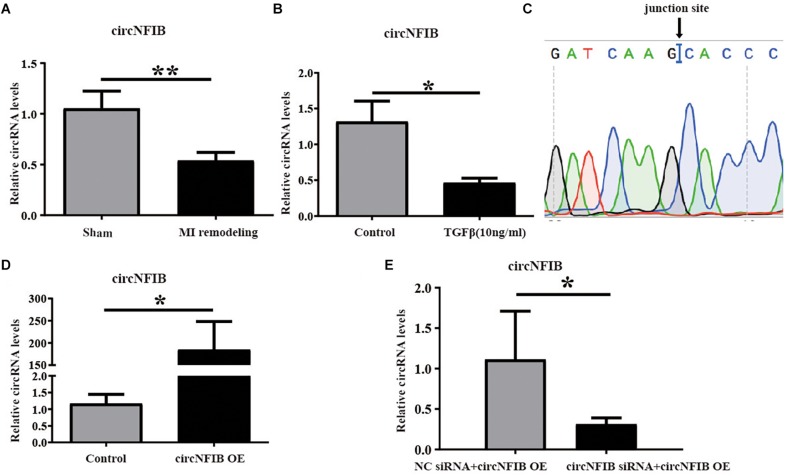
CircNFIB is decreased in cardiac fibrosis. **(A)** CircNFIB is decreased in ventricle samples from 21 days post-myocardial infarction mice (*n* = 5:6). **(B)** circNFIB is decreased in primary adult cardiac fibroblasts treated by TGF-β (*n* = 5:6). **(C)** Results of sequencing of divergent PCR products generated from circNFIB confirmed the head-to-tail junction point. **(D)** The transfection efficacy of circNFIB plasmid is confirmed by qRT-PCR (*n* = 6). **(E)** The transfection efficacy of circNFIB siRNA is confirmed by qRT-PCR (*n* = 4). ^*^p < 0.05, ^∗∗^*p* < 0.01 versus controls.

Previously, we have studied the essential role of miR-433 in regulating cardiac fibrosis (Tao et al., 2016). CircRNAs have been reported to act as miRNA sponges to regulate the function of miRNAs (Wang et al., 2016; Cai et al., 2019). This encourages us to investigate whether circRNAs can function as miR-433 sponge to modulate cardiac fibrosis. Firstly, we identified seven circRNAs (circBase: mmu_circ_0001140, mmu_circ_0004085, mmu_circ_0004087, mmu_circ_0001221, mmu_circ_0011794, mmu_circ_0000377, and mmu_circ_0004047) related to miR-433 in mouse heart samples by bioinformatics analysis. Next, we designed divergent primers for those circRNAs to amplify the back-splicing regions from mouse heart, and PCR products close to the predicted head-to-tail junction size would be subjected to Sanger sequence for further analysis. Then, we found that three out of seven circRNAs (circBase: mmu_circ_0011794, mmu_circ_0000377, and mmu_circ_0004047) had verified junction sites (Supplementary Figure S2). Using the Masson’s Trichrome staining approach, we have previously reported that heart samples from 3 weeks post-MI exhibited massive fibrosis (Tao et al., 2016). To further confirm which circRNA is involved in cardiac fibrosis, we explored the expression levels of these three circRNAs in heart samples from the 3-week post-MI model. Based on quantitative real-time polymerase chain reaction (qRT-PCR) analysis, we confirmed that only circNFIB (circBase: mmu_circ_0011794) was downregulated in heart samples from the 3-week post-MI mice (Figure 1A and Supplementary Figure S3). In addition, the expression of circNFIB was significantly reduced in TGF-β-treated cardiac fibroblasts (Figure 1B). These findings indicate a strong connection between the expression of circNFIB and cardiac fibrosis both *in vivo* and *in vitro*. The full length of circNFIB was 240 nucleotides and named based on its host gene NFIB, which is located in chromosome 4. Accurate overexpression construction of circNFIB was validated by a divergent primer amplified PCR product following Sanger sequencing (Figure 1C).

### Overexpression of circNFIB Attenuates Cardiac Fibroblast Proliferation Induced by TGF-β

**FIGURE 2:**
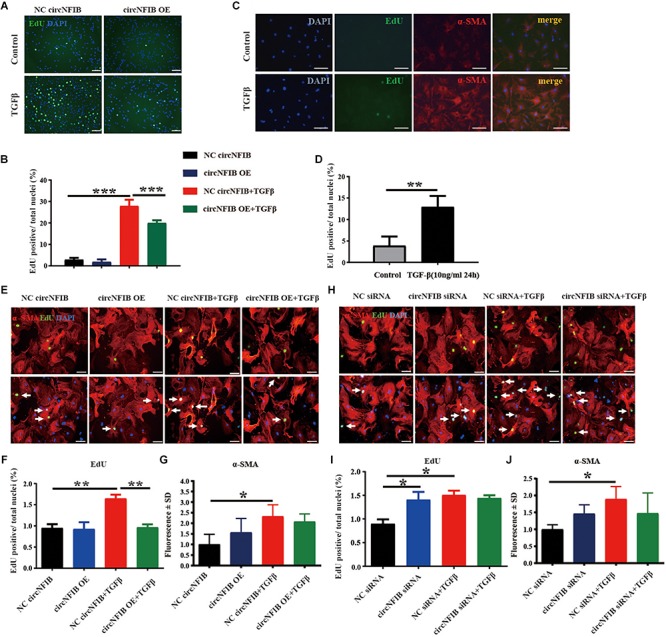
Overexpression of circNFIB attenuates cardiac fibroblast proliferation induced by TGF-β. **(A,B)** Forced expression of circNFIB decreases proliferation of NIH/3T3 cell stimulated by TGF-β, as evidenced EdU/α-SMA staining (*n* = 4). **(C,D)** Over-proliferation of primary adult fibroblasts is induced by TGF-β, as evidenced EdU/α-SMA staining (*n* = 5). **(E–G)** Forced expression of circNFIB does not have an effect on fibroblast activation but significantly decreased primary adult cardiac fibroblasts proliferation depending on TGF-β stimulation (*n* = 5). **(H–J)** Downregulation of circNFIB *via* siRNA promotes cardiac fibroblasts proliferation in the absence of TGF-β stimulation while having no statistical effect on α-SMA expression (*n* = 5). Scale bar: 50 μm. ^*^p < 0.05, ^∗∗^*p* < 0.01, ^∗∗∗^*p* < 0.001 versus controls.

Over-proliferation of cardiac fibroblasts is an essential process of cardiac fibrosis, contributing to both systolic and diastolic dysfunction during pathological remodeling (Wang et al., 2017; Park et al., 2018). TGF-β signaling is the major mechanism mediating fibroblast proliferation (Dobaczewski et al., 2011; Kong et al., 2014; Khalil et al., 2017; Felisbino and Mckinsey, 2018). In this study, overexpression and downregulation of circNFIB and its related NC were first transfected into primary adult cardiac fibroblasts, and transfection efficacy was confirmed by qRT-PCR (Figures 1D,E). Then, to evaluate the function of circNFIB in TGF-β stimulation, overexpression of circNFIB and relative NC were transfected into NIH/3T3 cell lines, and results showed that circNFIB overexpression attenuated cell proliferation as evidenced by the decreased ratio of EdU staining based on TGF-β stimulation (Figures 2A,B). To further confirm the role of circNFIB on fibroblast proliferation, primary adult cardiac fibroblasts were isolated from adult C57BL/6N mice and treated with TGF-β and over-proliferation of fibroblasts were induced (Figures 2C,D). Moreover, circNFIB overexpression attenuated cardiac fibroblast proliferation based on TGF-β stimulation (Figures 2E,F), while inhibition of circNFIB promoted fibroblast proliferation (Figures 2H,I). Downregulation of circNFIB failed to further enhance cardiac fibroblast proliferation in the presence of TGF-β stimulation (Figures 2H,I). In addition, neither upregulation nor inhibition of circNFIB had statistical effects on α-SMA expression (Figures 2G,J). Collectively, these data indicate that overexpression of circNFIB abates proliferation but not *trans*-differentiation of cardiac fibroblasts *in vitro*.

### CircNFIB Acts as a Competing Endogenous RNA for miR-433

**FIGURE 3:**
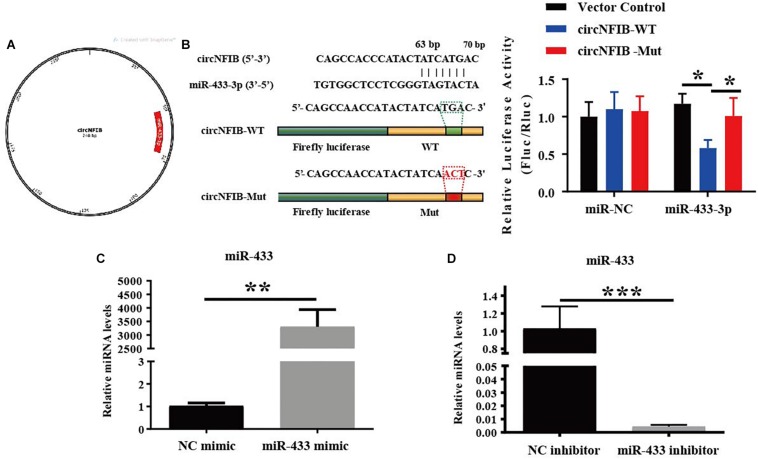
MiR-433 is identified as a direct target of circNFIB. **(A)** A schematic diagram displaying the putative binding site of miR-433 associated with circNFIB (processed using SnapGene). **(B)** Targetscan and Luciferase reporter assays identify miR-433 as a direct target of circNFIB (*n* = 6). **(C,D)** The transfection efficacy of miR-433 mimic (*n* = 6) or inhibitor (*n* = 4) is confirmed by qRT-PCR. ^*^p < 0.05, ^∗∗^*p* < 0.01, ^∗∗∗^*p* < 0.001 versus controls.

**FIGURE 4:**
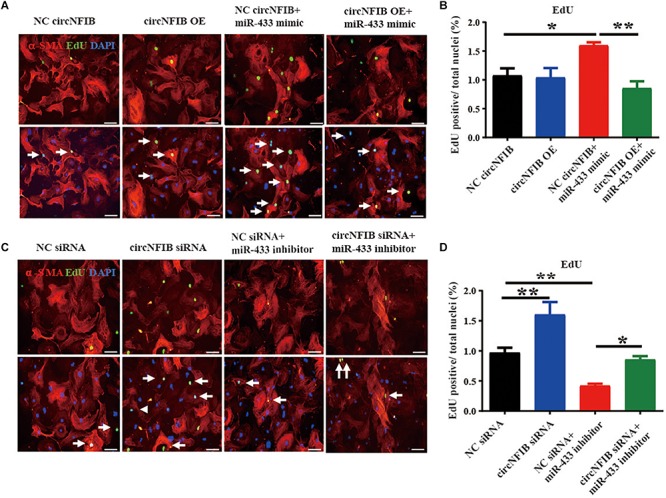
CircNFIB acts as a competing endogenous RNA for miR-433. **(A,B)** Overexpression of circNFIB attenuates the pro-proliferative effects of miR-433 mimic on cardiac fibroblasts, as evidenced EdU/α-SMA staining (*n* = 5). **(C,D)** Downregulation of circNFIB reverses the anti-proliferative responses of miR-433 inhibitor on cardiac fibroblasts, as evidenced EdU/α-SMA staining. Scale bar: 50 μm. ^*^p < 0.05, ^∗∗^*p* < 0.01, versus controls.

Previously, we have already shown that inhibition of miR-433 attenuated cardiac fibroblast proliferation and myofibroblast differentiation in murine post-MI models and in fibroblasts induced by TGF-β and AngII stimulation. In contrast, upregulation of miR-433 promoted cardiac fibrotic response (Tao et al., 2016). Therefore, we proposed that circNFIB may impact fibroblast proliferation through acting as a competing endogenous RNA for miR-433. RNAhybrid (Kruger and Rehmsmeier, 2006) and TargetScan (Agarwal et al., 2015) were used for miRNA recognition sequences on mouse circNFIB and revealed one putative miR-433 binding site (Figures 3A,B). Luciferase reporter assay revealed that miR-433 significantly inhibited luciferase activity for the wild-type 3′UTR construct for circNFIB but had no effect when the miR-433 binding site in the circNFIB was mutated, indicating a direct interaction between miR-433 and circNFIB (Figure 3B). To investigate whether the effects of circNFIB on fibroblast proliferation were mediated by miR-433, overexpression or inhibition of circNFIB and miR-433 was co-transfected into primary adult cardiac fibroblasts (Figures 3C,D). Our data illustrated that overexpression of circNFIB could abate the pro-proliferative effects of the miR-433 mimic on cardiac fibroblasts as evidenced by decreased EdU-positive cells (Figures 4A,B). Furthermore, downregulation of circNFIB *via* siRNA also reversed the anti-proliferative effects of the miR-433 inhibitor as identified by an increase in EdU staining (Figures 4C,D).

**FIGURE 5:**
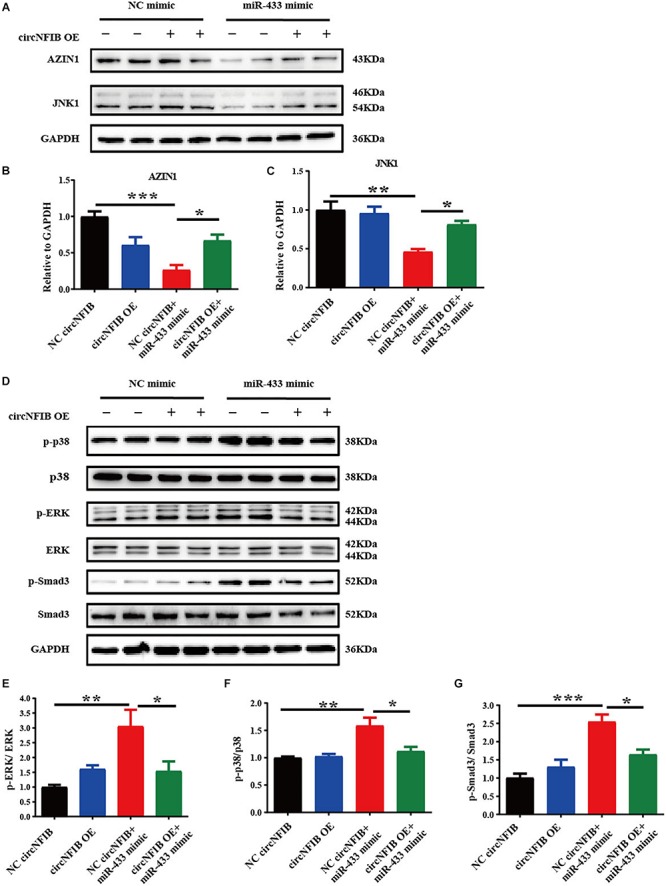
CircNFIB reverses the expression level of target genes and downstream signaling pathways of miR-433. **(A–C)** Overexpression of circNFIB reverses expression level of miR-433 target genes AZIN1 and JNK1 by Western blot analysis (*n* = 4). **(D–G)** Overexpression of circNFIB reverses downstream signaling pathways of miR-433, as evidenced by ratio of p-ERK/ERK, p-p38/p38, and p-Smad3/Smad3 by Western blot analysis (*n* = 4). ^*^p < 0.05, ^∗∗^*p* < 0.01, ^∗∗∗^*p* < 0.001 versus controls.

As AZIN1 and JNK1 were previously confirmed as target genes of miR-433, further researches demonstrated that knockdown of AZIN1 could promote proliferation and differentiation of cardiac fibroblasts into myofibroblasts accompanied by an activation of the TGF-β–Smad3 signaling pathway. Besides AZIN1, reduction of JNK1 was responsible for the pro-fibrotic effects by activation of ERK and p38 kinase, in parallel with the activation of Smad3 (Tao et al., 2016). Therefore, we further examined the modulatory effects of circNFIB on miR-433 target genes and downstream signaling pathways. Our data showed that the inhibition of AZIN1 and JNK1 by the miR-433 mimic was reversed by overexpression of circNFIB (Figures 5A–C). Moreover, upregulation of circNFIB also attenuated the activation of p38, ERK kinases, and the Smad3 signaling pathway as evidenced by the decreased ratio of p-p38/p38, p-ERK/ERK, and p-Smad3/Smad3 (Figures 5D–G). However, circNFIB failed to have an additive effect on target genes and downstream signaling pathways independent of miR-433 (Figures 5A–G). These data suggest that circNFIB is an endogenous competing sponge of miR-433 mediating its effects in cardiac fibrosis.

## Discussion

Despite the high incidence of mortality and morbidity related to cardiac fibrosis, no fibrotic strategies are available under the current treatment regimens recommended by the official guidelines such as angiotensin-converting enzyme inhibitors (ACEIs), angiotensin receptor blockers (ARBs), and aldosterone synthase inhibitors (Ghosh et al., 2013; Weber et al., 2013; Xie et al., 2013). Thus, targeting the direct molecular mediators holds promise to develop therapeutic interventions. Recently, novel therapeutic strategies such as non-coding RNAs (ncRNAs) (Wang et al., 2018), exosomes (Bellin et al., 2019), and exercise (Cipryan, 2018; Randers et al., 2018) have been reported to perform beneficial effects against diverse cardiovascular diseases. The roles of circRNAs in cardiovascular diseases including cardiac fibrosis have attracted much attention (Bei et al., 2018).

CircRNAs are endogenous, abundant, and stable ncRNAs with diverse action modes formed by back-splicing events (Zeng et al., 2017). The mechanisms for circRNA function in the physiological or pathological heart have not been well elucidated clearly, especially in cardiac fibrosis. The most reported function pattern for circRNA is acting as a miRNA sponge and forming the circRNA–miRNA–mRNA axis (Chen, 2016; Du et al., 2017). It has been suggested that dysregulated circRNAs, such as circRNA_010567 and circRNA_000203, contribute to cardiac fibrosis by sponging miR-141 and miR-26b-5p, respectively (Tang et al., 2017; Zhou and Yu, 2017). Our previous data revealed that overexpression of miR-433 promoted cardiac fibroblast proliferation and their differentiation into myofibroblasts both *in vivo* and *in vitro*, while knockdown of miR-433 inhibited fibrotic effects by targeting AZIN1 and JNK1. Moreover, the protective effects of miR-433 inhibition against cardiac fibrosis were confirmed by administration of exogenous miR-433 sponge *via* cardiotropic AAV9. Therefore, we hypothesized that circNFIB may act as a miR-433 endogenous competing sponge regulating cardiac fibrosis. CircNFIB was derived from the exon regions of the *Nfib* gene. *Nfib*-encoded protein nuclear factor 1 B-type (NF1-B) belongs to the CTF/NF-I family and is relatively highly expressed in mouse heart^1^. However, it has no relevant reports in the heart field so far. As a CCAAT-box-binding transcription factor, NF1-B is involved in the biological process of DNA replication, transcription, and transcription regulation. Therefore, it is possible that NF1-B itself takes an important part in the heart, and we cannot rule out the possibility that circNFIB and Nfib mRNA, even protein NF1-B, may have functional relevance, but we have not studied it here. Of course, further efforts to explore the functional difference and correlation between NFIB mRNA and circ-NFIB should be of great significance.

Activation of multiple key fibrogenic pathways has been shown to contribute to the progression of fibrosis (Piccoli et al., 2017; Xie et al., 2018; Martinez-Martinez et al., 2019). Proliferation of the resident fibroblasts is one of the major mechanisms. This concept is supported by a recent fate mapping study that revealed that the excessive proliferation of fibroblasts may result in fibroblast accumulation after pressure overload (Moore-Morris et al., 2014). In our present study, knockdown of circNFIB promoted cardiac fibroblast proliferation as exhibited by the increased number of EdU-positive cells. Notably, the overexpression of circNFIB prevents the increase in cell proliferation in TGF-β induced fibrosis in both NIH/3T3 cell lines and primary adult cardiac fibroblasts. However, neither overexpression nor inhibition of circNFIB has an additional role on α-SMA expression, indicating that circNFIB may act as a novel anti-proliferative effector *in vitro*; however, its role in cardiac fibrosis *in vivo* remains largely unknown. In addition, the function of circNFIB in different models of cardiac fibrosis is still uncertain and necessitates future study.

In the present study, miR-433 was confirmed to be a direct target of circNFIB by luciferase reporter assay. Functional analysis showed that upregulation of circNFIB attenuated the proliferation of cardiac fibroblasts while downregulation of circNFIB reversed the anti-proliferative effects of the miR-433 inhibitor. Our previous research demonstrated that miR-433 performs pro-fibrotic effects by inhibiting its target genes AZIN1 and JNK1. Knockdown of AZIN1 could promote proliferation and differentiation of cardiac fibroblasts into myofibroblasts accompanied by an activation of the TGF-β–Smad3 signaling pathway. Furthermore, reduction of another target gene JNK1 was also responsible for the pro-fibrotic effects of miR-433 in cardiac fibroblasts accompanied by activation of ERK and p38 kinase signaling pathways (Tao et al., 2016). Currently, we suggest that overexpression of circNFIB reversed the effects of AZIN1 and JNK1 and downstream signaling pathways including ERK, p38, and Smad3 by the miR-433 mimic, suggesting that circNFIB may serve as an endogenous competing sponge of miR-433 regulating cardiac fibrosis. However, there are still some questions that need to be answered in future studies. As our results show, overexpression of circNFIB fails to influence fibroblast proliferation independent of TGF-β and circNFIB also fails to have an additive effect on target genes and downstream signaling pathways independent of miR-433. Furthermore, neither overexpression nor inhibition of circNFIB has additional roles on cardiac fibroblast *trans*-differentiation. However, in our previous study, inhibition of miR-433 attenuated both proliferation and differentiation of cardiac fibroblasts. This inconsistent role observed between circNFIB and miR-433 on cardiac fibrosis could be attributed to two points. First, a single miRNA can be regulated by multiple factors, such as circRNAs, long ncRNAs, or other proteins (Pu et al., 2019). In this case, circNFIB may act as only one regulator of miR-433. There should be other upstream factors modulating the function of miR-433. Second, although our research here is limited to studying the regulatory role of the circNFIB–miR-433 axis, circRNAs may act as sponges of different miRNAs as previous studies demonstrated (Zheng et al., 2016). Besides miRNA sponges, circRNAs are also found to bind to RNA-binding proteins (RBPs) or regulate gene transcription (Li et al., 2015; Du et al., 2017). The diversity regulatory mechanisms of circRNAs indicate that circNFIB may also have other potential functioning mechanisms that need further in-depth investigation in the future.

## Conclusion

We have discovered that circNFIB expression is significantly decreased in heart samples from mice post-MI and TGF-β-treated cardiac fibroblasts. Functionally and mechanistically, overexpression of circNFIB attenuates cardiac fibroblast proliferation depending on TGF-β stimulation. Furthermore, miR-433 is identified as a direct target of circNFIB, and circNFIB could rescue the effects of target genes and downstream signaling pathways of miR-433. Taken together, our study indicates that the circNFIB–miR-433 axis may function as a novel therapy for cardiac fibrosis.

## Data Availability

The raw data supporting the conclusions of this manuscript will be made available by the authors, without undue reservation, to any qualified researcher.

## Ethics Statement

All animals were purchased from the Cavens Laboratory Animal (Changzhou, China) and raised at the Experimental Animal Center of Shanghai University (Shanghai, China). All the procedures with animals were in accordance with the guidelines of the National Institutes of Health (No. 85-23, revised in 1996). The protocols in this study were approved by the ethical committees of School of Life Sciences, Shanghai University.

## Author Contributions

LW and LT designed the study, conducted all the experiments, and drafted the manuscript. YZ, WP, TY, XM, and ZJ conducted the experiments and analyzed the data.

## Conflict of Interest Statement

The authors declare that the research was conducted in the absence of any commercial or financial relationships that could be construed as a potential conflict of interest. The handling Editor declared a past co-authorship with one of the authors LT.

## Footnotes

1 https://www.ncbi.nlm.nih.gov/gene/18028
