# Supplementary material for: Upregulation of Circular RNA CircNFIB Attenuates Cardiac Fibrosis by Sponging miR-433
Source: Front Genet. 2019 Jun 20;10:564. doi: 10.3389/fgene.2019.00564 (PMC6611413; doi:10.3389/fgene.2019.00564)

Supplemental Figure1: Uncropped images of western blot of Figure 5A, 5D

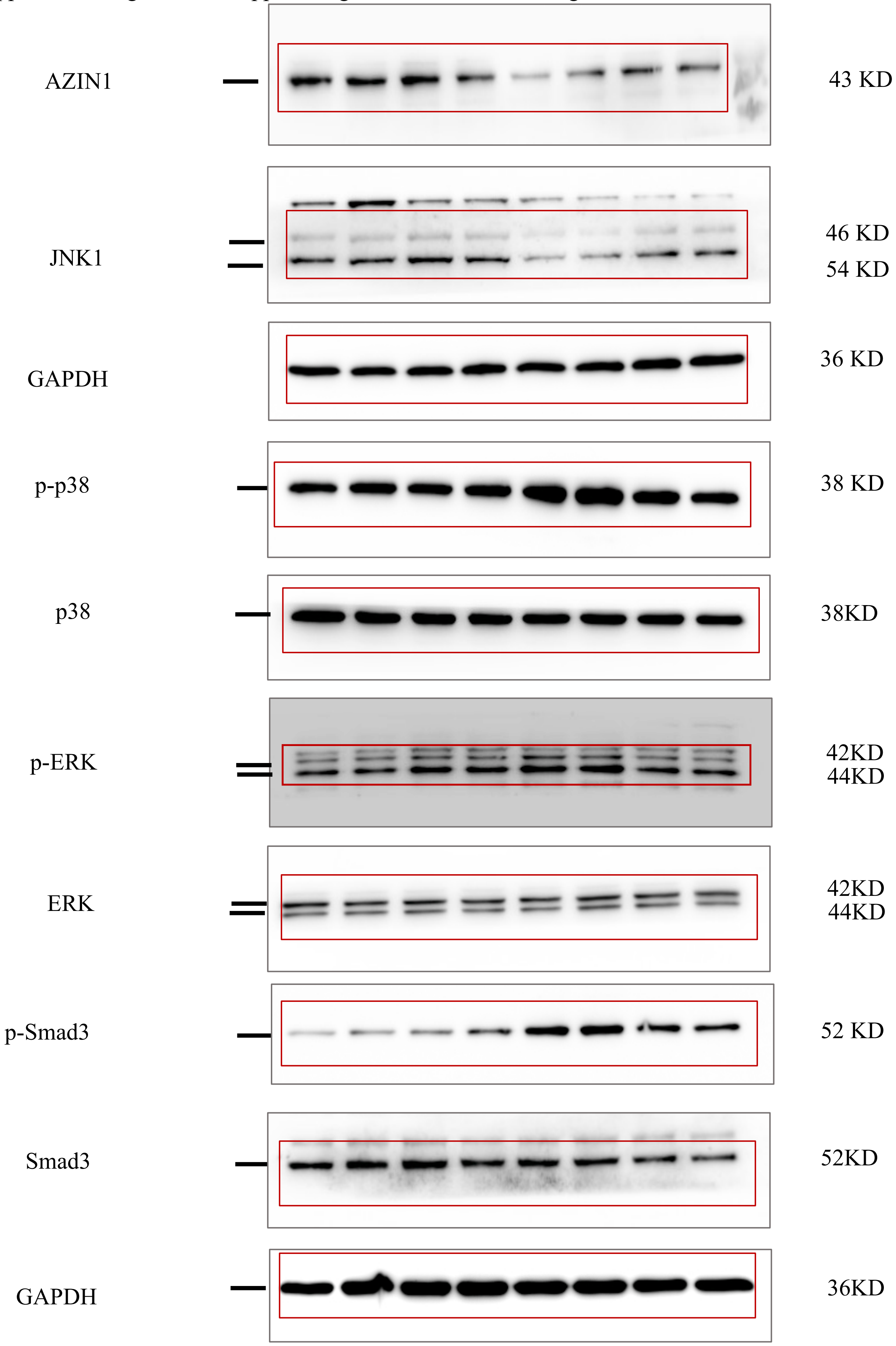

Supplemental Figure 2: Sanger sequence analysis identified mmu_circ_0011794, mmu_circ_0000377 and mmu_circ_0004047 had verified junction sites in mouse heart.

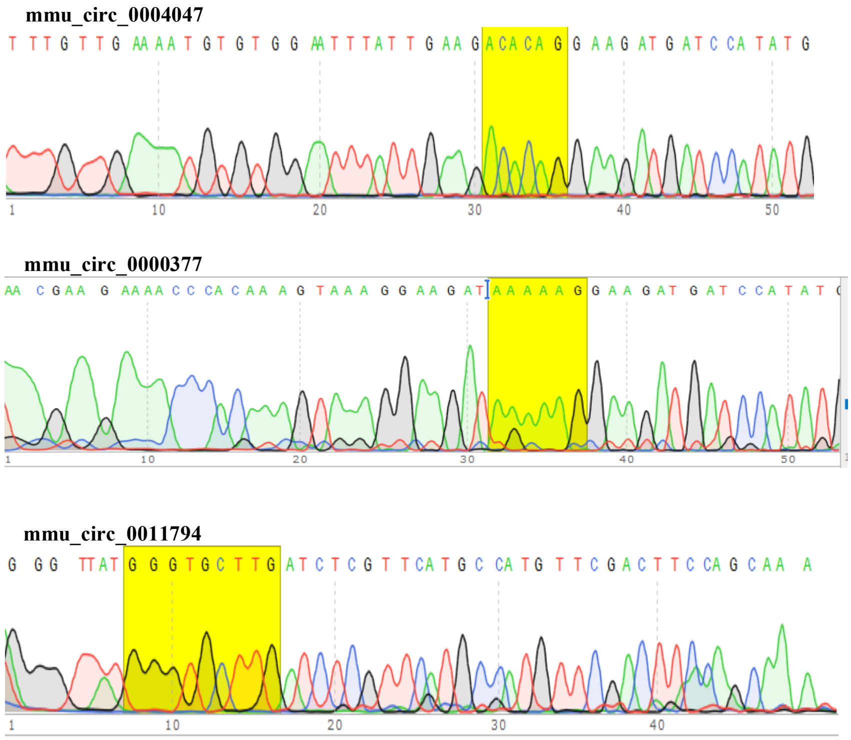

Supplemental Figure 3: The expression levels of mmu_circ_0000377 and mmu_circ_0004047 in heart samples from 3 weeks post-MI model. n=5.

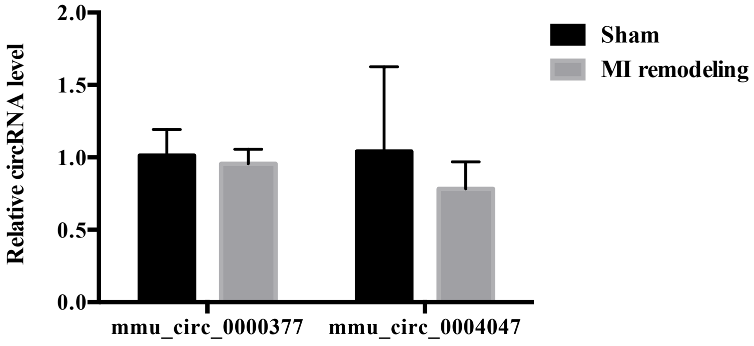

Supplement: Supplementary file 1 [file Data_Sheet_1.PDF]
